# Structure-function analysis of the heme-binding WWD domain in the bacterial holocytochrome *c* synthase, CcmFH

**DOI:** 10.1128/mbio.01509-23

**Published:** 2023-11-06

**Authors:** Amber L. Grunow, Susan C. Carroll, Alicia N. Kreiman, Molly C. Sutherland

**Affiliations:** 1 Department of Biological Sciences, University of Delaware, Newark, Delaware, USA; University of Michigan-Ann Arbor, Ann Arbor, Michigan, USA; Michigan State University, East Lansing, Michigan, USA

**Keywords:** cytochrome biogenesis, heme transport, cytochromes, heme

## Abstract

Heme trafficking is a fundamental biological process, yet its direct study has been hampered due to heme’s tight intracellular regulation, heme cytotoxicity, and the transient nature of trafficking. The bacterial System I and System II cytochrome *c* biogenesis pathways are developing into models to interrogate heme trafficking mechanisms, as they function to transport heme from inside to outside the cell for attachment to apocytochrome *c*. Cytochromes *c* require heme for folding and to function in the context of electron transport chains for critical cellular functions, such as respiration. We focus on System I, comprised of eight membrane proteins, CcmABCDEFGH, proposed to function in two steps: CcmABCD mediates the transfer of heme and attachment to CcmE. HoloCcmE chaperones heme to CcmFH for attachment to apocytochrome *c*. While CcmFH is known to be the holocytochrome *c* synthase, the mechanism of heme interaction and positioning for attachment to apocytochrome *c* remains to be elucidated. A comprehensive structure-function analysis of the conserved WWD domain in CcmF was undertaken utilizing alanine-scanning and cysteine-scanning, revealing residues critical for CcmF’s synthase function and residues required for interaction with the 2- and 4-vinyls of heme. This analysis demonstrates for the first time that the CcmF WWD domain directly interacts with heme and that heme interactions within this domain are required for attachment to apocytochrome *c*. This in-depth interrogation of heme binding now allows for comparison across cytochrome *c* biogenesis proteins CcmF, CcmC, and CcsBA, revealing common mechanisms of heme interaction in these heme trafficking pathways.

**IMPORTANCE:**

Heme is an essential co-factor for proteins involved with critical cellular functions, such as energy production and oxygen transport. Thus, understanding how heme interacts with proteins and is moved through cells is a fundamental biological question. This work studies the System I cytochrome *c* biogenesis pathway, which in some species (including *Escherichia coli*) is composed of eight integral membrane or membrane-associated proteins called CcmA-H that are proposed to function in two steps to transport and attach heme to apocytochrome *c*. Cytochrome *c* requires this heme attachment to function in electron transport chains to generate cellular energy. A conserved WWD heme-handling domain in CcmFH is analyzed and residues critical for heme interaction and holocytochrome *c* synthase activity are identified. CcmFH is the third member of the WWD domain-containing heme-handling protein family to undergo a comprehensive structure-function analysis, allowing for comparison of heme interaction across this protein family.

## INTRODUCTION

Nearly all organisms encode cytochromes *c*, diverse proteins that facilitate critical cellular functions such as respiration, photosynthesis, and detoxification of radical oxygen species. All cytochromes *c* require the attachment of a heme co-factor at a conserved CysXxxXxxCysHis motif via covalent thioether bond formation between the reduced cysteine thiols of the CXXCH motif and heme vinyl groups. Heme transport and positioning for attachment to CXXCH are accomplished by three dedicated protein pathways for cytochrome *c* biogenesis: System I (CcmA–H; α,γ Proteobacteria; plant and protozoal mitochondria; Archaea), System II (CcsBA; Gram positive; cyanobacteria; chloroplasts; ε Proteobacteria), and System III (HCCS; eukaryotic mitochondria) (reviewed in references 1–8).

All three cytochrome *c* biogenesis pathways function to interact with and position heme for attachment to apocytochrome *c*, albeit by different mechanisms. In Systems I and II, the pathways are also proposed to move heme across the bacterial membrane, thus these pathways are developing into important model systems to probe general mechanisms of heme binding and transport. Despite recent advances in the development of heme sensors for the intracellular heme pool (9–14), directly mapping pathways for heme transport remains an important facet to probing this fundamental biological process. For example, a novel cysteine/heme crosslinking approach was used to identify discrete heme interaction domains in System I proteins CcmC and CcmE, as well as System II, CcsBA (15, 16). Molecular details of heme interaction domains and models of heme transport have been expanded by recent cryo-EM structures of CcmABCD and CcsBA (17–19). However, many questions remain regarding heme transport and binding in the bacterial cytochrome *c* biogenesis pathways. Here, we focus on the System I pathway, specifically the holocytochrome *c* synthase, CcmFH.

**Fig 1:**
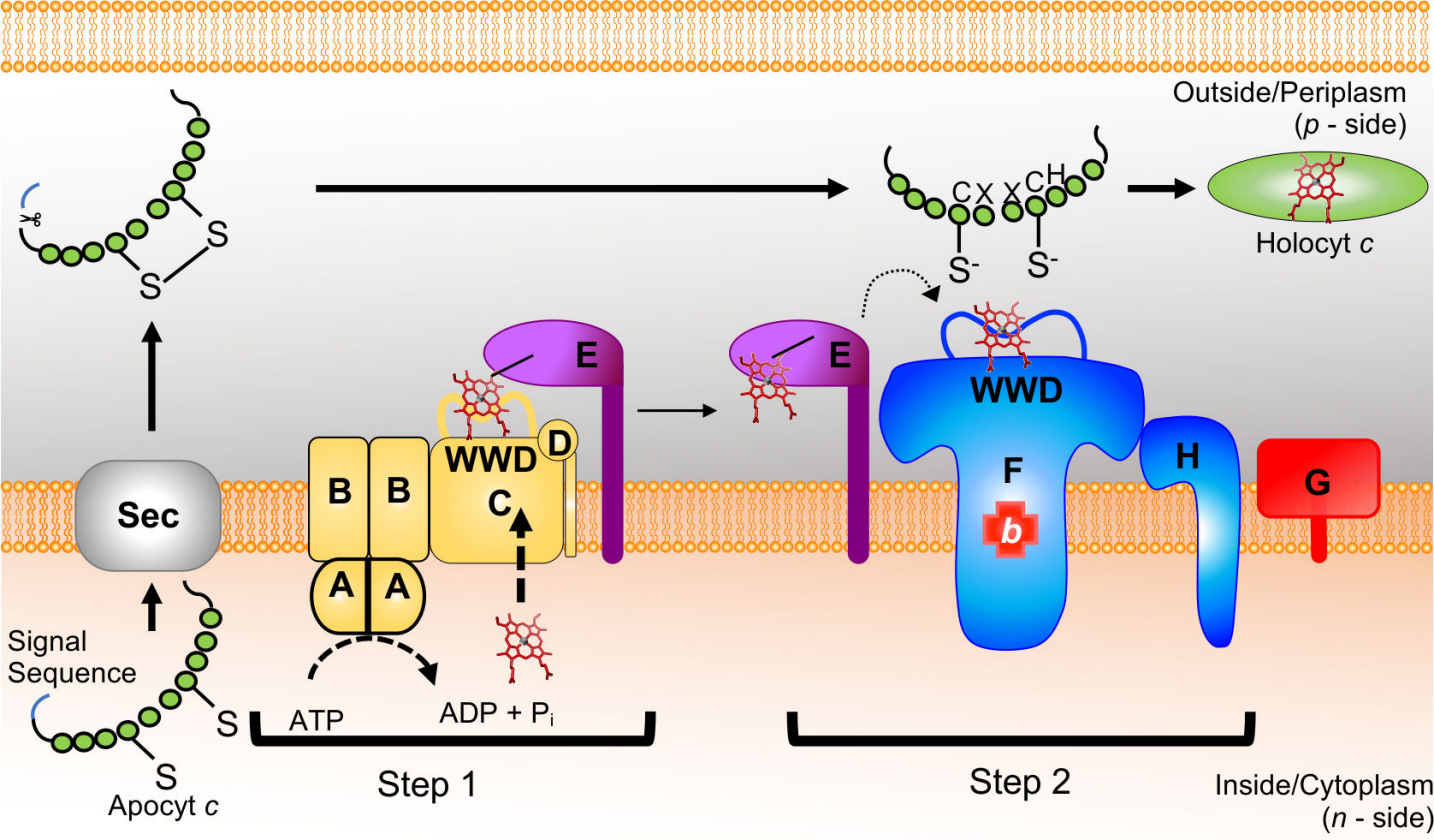
Schematic of *E. coli* System I bacterial cytochrome *c* biogenesis pathway. System I is composed of eight proteins, CcmABCDEFGH, that function in two steps. First, heme is transported to the CcmC WWD domain, and CcmABCD (gold) mediates the covalent attachment of heme to CcmE (purple). HoloCcmE is released via CcmA-dependent ATP hydrolysis and is proposed to chaperone heme (indicated by dotted arrow) to the holocytochrome *c* synthase, CcmFH (blue). CcmFH attaches heme to apocytochrome *c* (green) at a conserved CXXCH motif via thioether bond formation between the heme vinyl groups and the cysteine thiols. CcmG (red) acts as a thioredoxin.

The System I pathway, encoded by *Escherichia coli*, is composed of eight cytochrome *c* maturation proteins (CcmABCDEFGH). In several other bacterial species, System I is composed of nine integral membrane proteins (CcmABCDEFGHI), where the *E. coli ccmH* is encoded as two open reading frames designated as *ccmH* and *ccmI* (reviewed in references 4, 6, 20). System I is proposed to function in two steps to transport heme across the inner membrane to the holocytochrome *c* synthase for attachment to apocytochrome *c* (Fig. 1). First, heme is transported from the site of synthesis inside the cell to the periplasmic CcmC WWD domain (16, 18), whereby CcmABCD (18, 21–23) mediates heme attachment to CcmE (24, 25). HoloCcmE is released by ATP hydrolysis via CcmABCD, which functions as an ABC transporter release complex (18, 21). Next holoCcmE is proposed to chaperone heme to CcmFH, the holocytochrome *c* synthase, for attachment to apocytochrome *c* (26, 27) (Fig. 1).

**Fig 2:**
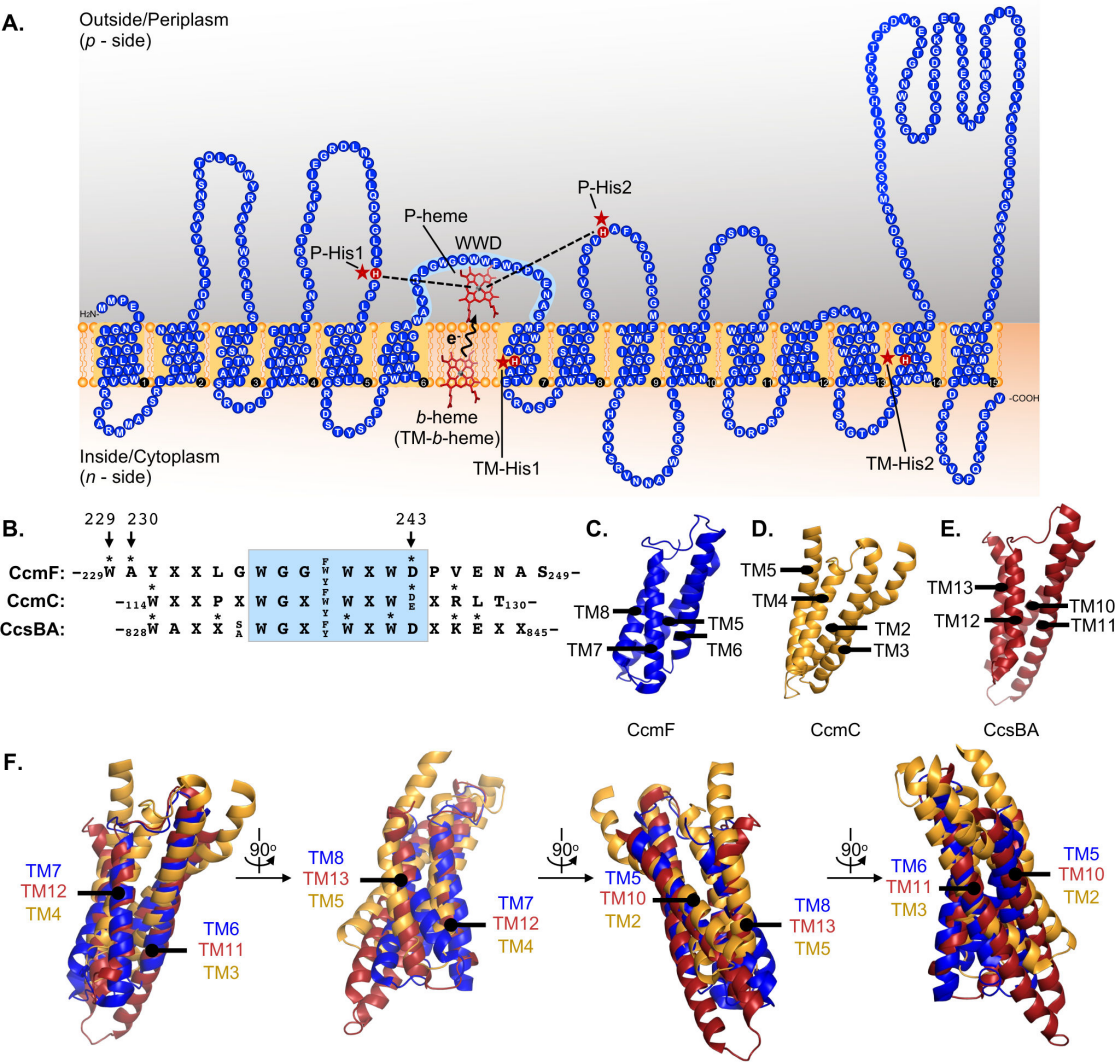
The CcmF WWD domain. (**A**) Based on *Thermus thermophilus* crystal structure [(32); PDB 6ZMQ], an updated topology of *E. coli* CcmF, containing 15 transmembrane domains, is proposed. Conserved features of CcmF are indicated: TM-*b-*heme liganded by TM-His1/TM-His2, WWD domain, and P-heme liganded by P-His1/P-His2. (**B**) Sequence conservation of the WWD domain in *E. coli* System I proteins CcmF (W229-S249), CcmC (W114-T130), and *H. hepaticus* System II protein CcsBA (W828-X845). Variable residues—“X,” conserved and semi-variant residues are indicated. Residues that form a cysteine/heme crosslink are denoted with an asterisk and in the case of CcmF are denoted by residue number. (**C–E**) The WWD “core region” of CcmF (C) (blue, PDB: 6ZMQ), CcmC (D) (gold, “open” conformation, no heme in the WWD domain, PDB: 7F03), and CcsBA (E) (red, “closed” conformation, no heme in the WWD domain PDB: 7S9Z) are shown and contain a similar architecture of four transmembrane domains surrounding the periplasmic WWD domain. (**F**) Overlays of the WWD transmembrane cores from CcmF, CcmC, and CcsBA are shown with 90° rotation to display variations in structural alignment.

Key features of CcmFH have been elucidated by genetic and biochemical analyses. These include CcmF’s requirement for interaction with CcmH for holocytochrome *c* synthase function (22, 26, 28–30), the presence of a stably bound transmembrane *b*-heme (22, 31, 32) (here designated as TM-*b-*heme) liganded by two conserved histidines (TM-His1, 2) (31), a conserved WWD domain and two conserved periplasmic histidines (P-His1, 2) (Fig. 2A) (22, 26, 31). The conserved WWD domain is a tryptophan-rich region encoded in heme-handling proteins (HHPs) such as CcmF, CcmC, and CcsBA (System II) (33). The WWD domain directly interacts with heme and is required for heme attachment in CcmC (16, 18) and CcsBA (15, 17), but its role in CcmF has not been elucidated. Here, we undertake a comprehensive structure-function analysis of the CcmF WWD domain to determine its role in System I holocytochrome *c* synthase function and to further map the heme trafficking pathway of System I. We identify WWD residues that are required for CcmFH synthase function, as well as residues that are directly required for heme interaction, thus the WWD domain localized heme is designated here as P-heme due to its periplasmic localization and ligation by P-His1, 2. This comprehensive analysis of CcmF allows for a comparison of WWD heme interaction across bacterial cytochrome *c* biogenesis proteins, revealing a conserved mechanism of heme interaction, as well as key differences.

## RESULTS

### Topology of *E. coli* CcmF

Recently, a crystal structure of *Thermus thermophilus* CcmF was determined (32). Although this structure lacks the CcmF functional protein partner CcmH, it still provides valuable insights into CcmF. Here, we focus on *E. coli* CcmF, which has ~36% sequence identity to *T. thermophilus* CcmF (UniProt BLAST [34]; Fig. S1A). Utilizing AlphaFold2 AI structure prediction software (35, 36), a predicted structure of *E. coli* CcmF was generated (Fig. S1B). Alignment of the *T. thermophilus* crystal and *E. coli* AlphaFold2 CcmF structures indicates general conservation of structural architecture (Fig. S1B), despite low sequence identity. The *T. thermophilus* CcmF crystal (32), *E. coli* AlphaFold2 predicted structure (Fig. S1B), and a recent AF2Complex prediction of CcmFH (37) each contain 15 transmembrane domains (TMD). Therefore, an updated topology schematic for *E. coli* CcmF with the stable TM-*b-*heme, conserved TM-His1/2, P-His1/2, and WWD domain with P-heme is shown (Fig. 2A).

### Insights into the WWD domain of the heme-handling protein family

Despite these advances in structural knowledge, a major knowledge gap remains in the molecular mechanisms of CcmF synthase function, in particular, the binding and positioning of heme prior to attachment to apocytochrome *c*. CcmF is a member of the HHP family, which also includes CcmC and CcsBA (33, 38), all of which encode a tryptophan-rich region, known as the WWD domain, flanked by two conserved periplasmic histidines (33, 38) (Fig. 2A and B). The WWD domains of CcmC and CcsBA have been shown to directly bind heme via cysteine/heme crosslinking (15, 16), and heme binding was resolved in subsequently determined cryo-EM structures (17, 18). A “core region” consisting of the four transmembrane domains surrounding the WWD domain was previously defined in HHPs CcmC (16, 18) (Fig. 2D) and CcsBA (15, 17) (Fig. 2E). This “core region” is defined as TMD 5–8 in CcmF (32) (Fig. 2C). These core regions were overlayed (Fig. 2F) utilizing the structural conformations of CcmC (PDB: 7F03) and CcsBA (PDB: 7S9Z) that correspond with the CcmF structure (PDB: 6ZMQ). Since the CcmF crystal structure lacks heme in the WWD domain, we wanted to determine if structural conservation of the core region is retained among these proteins. While the general architecture of the “core region” consisting of the WWD domain surrounded by four core TMDs (Fig. 2C through E) is retained, the positioning of the TMDs differs between the three proteins (Fig. 2F; Fig. S2). This is unsurprising as each of the HHP family members is predicted to have different mechanisms for the reception of P-heme into the WWD domain (e.g., CcmF is proposed to receive heme from CcmE in the periplasm with the TMDs stabilized by the TM-*b*-heme, while CcsBA is predicted to transport heme across the inner membrane resulting in conformational changes to the TMDs during transport), thus may have different structural requirements (17, 18, 32).

### The conserved WWD domain is required for CcmF holocytochrome *c* synthase function

**Fig 3:**
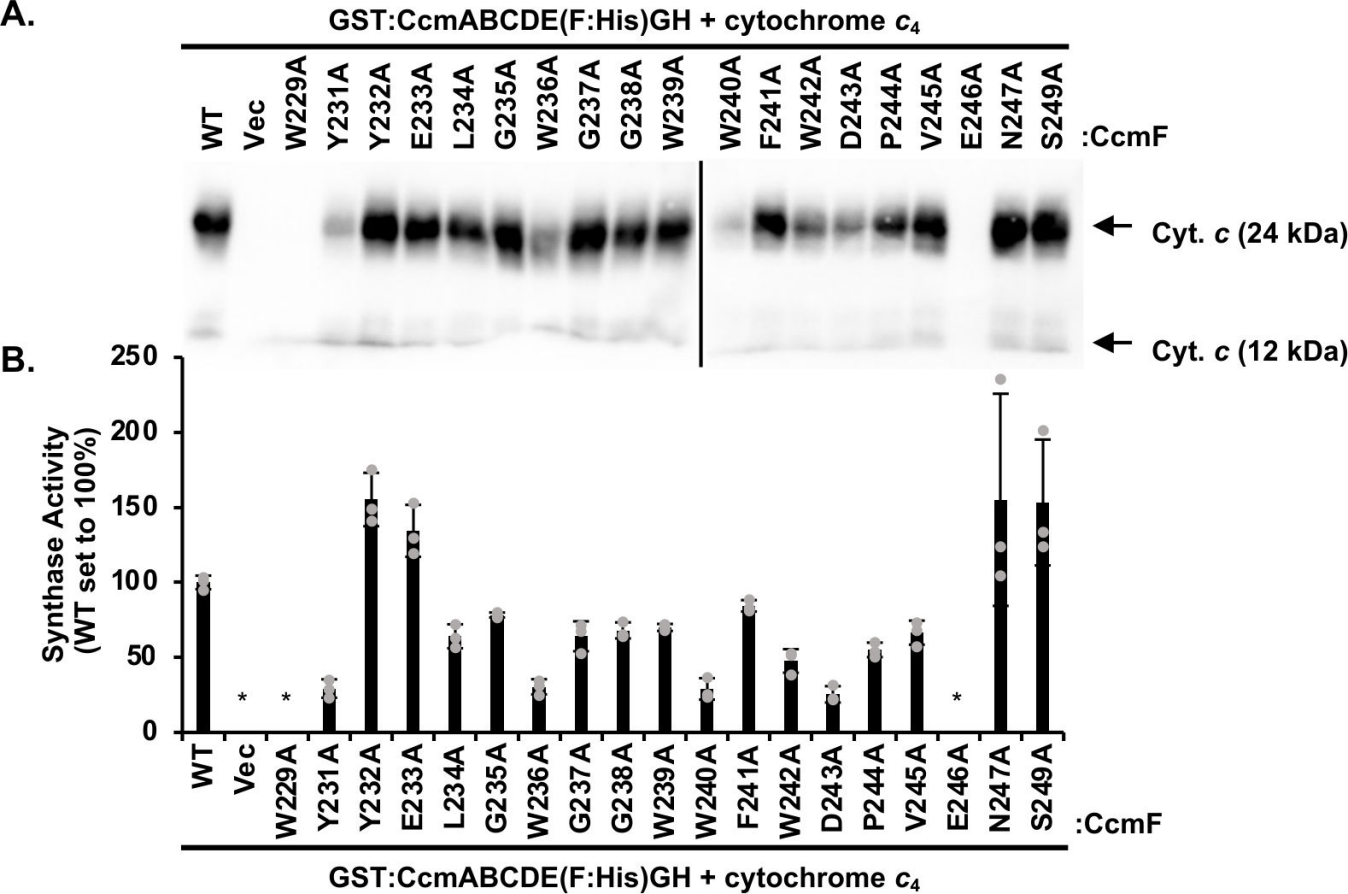
The WWD domain is required for CcmFH holocytochrome *c* synthase function. (**A**) CcmF:His wild-type or alanine variants were engineered in the context of the full System I pathway (CcmA–H) and recombinantly co-expressed with cytochrome *c*_4_:His in *E. coli* Δ*ccm*. Synthase activity was analyzed by cell lysis, separation of 50 µg total lysate by SDS-PAGE, transfer to a nitrocellulose membrane, and relative amount of cytochrome *c* produced was determined by an ECL-based heme stain. Three biological replicates, each containing three technical replicates, were performed. Representative samples are shown. (**B**) Quantitation of the CcmF:His wild-type and alanine variant synthase function. WT is normalized to 100% function. Error bars show the standard deviation from the mean, and dots indicate individual data points. Representative biological replicate is shown. The asterisk indicates below detectable limit of heme stain.

Previous work has determined that the WWD domain in the System I protein CcmC (16, 23) and System II proteins, CcsBA (15, 39–42), is required for protein function, specifically heme attachment to CcmE or apocytochrome *c*. However, the role of the CcmF WWD domain has not been systematically analyzed. Here, 19 single amino acid alanine substitutions were engineered in the CcmF WWD domain (W229–S249) (see Fig. 2B, CcmF) on a plasmid containing the complete System I pathway [GST:CcmABCDE(F:6×His)GH]. Note that two CcmF WWD residues already encode an alanine (A230 and A248), and thus are not included in this analysis. To determine if specific WWD domain residues were critical for CcmF holocytochrome *c* synthase function, alanine variants were recombinantly co-expressed with *Bordetella pertussis* cytochrome *c*_4_ in *E. coli* Δ*ccm* (RK103), and levels of cytochrome *c* biogenesis were assessed via an enhanced chemiluminescence (ECL)-based heme stain (Fig. 3). Two variants were deficient for CcmF synthase function (W229A and E246A), four variants had severely impaired synthase function (<30% of wild type [WT]) (Y231A, W236A, W240A, and D243A), eight variants retained partial synthase function (30%–80% of WT) (L234A, G237A, G238A, W239A, F241A, W242A, P244A, and V245A), while five retained wild-type levels of function (>80%) (Y232A, E233A, G235A, N247A, and S249A) (Fig. 3A and B; Fig S3). This analysis demonstrates that W229 and E246 are required for the synthase function of CcmF. These residues are among those conserved within the WWD domain of the HHP family (see Fig. 2B) and were also shown to be required for CcsBA synthase function (17), indicating similarities in mechanisms of heme attachment to apocytochrome *c* between the bacterial holocytochrome *c* synthases.

### The conserved WWD domain is not required for CcmF protein:protein interactions

**Fig 4:**
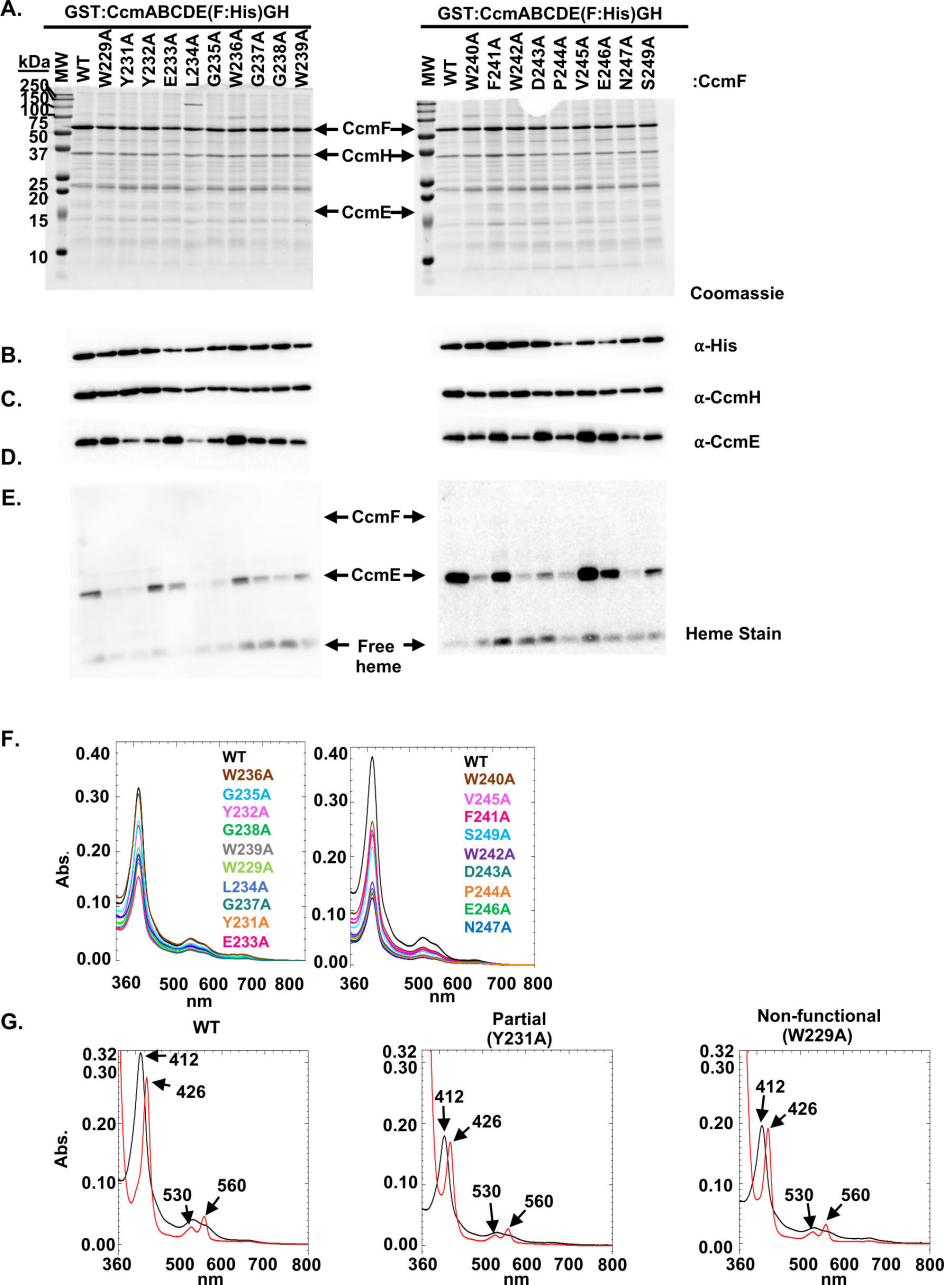
The CcmF WWD domain is not required for protein-protein interactions. CcmF:His wild-type or alanine variants were affinity purified, 5 µg of protein was separated by SDS-PAGE, and analyzed for (**A**) protein stability, (**B**) presence of His tag, (**C**) co-purification with CcmH, (**D**) co-purification with CcmE, and (**E**) presence of holoCcmE and *b*-type heme. Data (**A–E**) are representative of three independent purifications. (**F**) Heme co-purification was assayed via the heme Soret (410 nm) utilizing 50 µg of purified protein. Data are representative of three independent purifications. (**G**) UV-vis spectra of 50 µg of as-purified (black) and reduced (red) spectra of WT and representative CcmF WWD alanine variants from different functional classes.

To determine if the CcmF WWD domain plays a role in known interactions with CcmH (22, 28–30) or CcmE (27), a biochemical analysis of the WWD alanine variants was undertaken. Variants were affinity purified via a C-terminal 6×His affinity tag on CcmF. All CcmF WWD alanine variants were stable (Fig. 4A), contained the 6×His tag (Fig. 4B), co-purified with CcmH (Fig. 4C), and with holoCcmE (Fig. 4D and E). Thus, the observed defects in cytochrome *c* biogenesis were not due to disruption of protein subcomplexes known to be required for CcmFH synthase function (26, 27).

UV-vis spectral analysis was performed to determine if the CcmF WWD alanine variants had a defect in heme co-purification or disruption of the heme environment. CcmF co-purifies with a stable TM-*b-*heme liganded by TM-His1 and TM-His2 at a 1:1 stoichiometry (22, 31) (see Fig. 2A), and the majority of the heme signal in UV-vis spectral analysis is due to this TM-*b*-heme. First, overall levels of heme co-purification were analyzed via the Soret peak (~410 nm), which can be used to compare relative amounts of heme (Fig. 4F). All variants co-purified with heme, with no clear deficiencies evident across three independent purifications.

Next, UV-vis spectral analysis of as-purified and reduced spectra was determined. The CcmF WT and WWD alanine variants showed a characteristic shift in the Soret peak from 410 nm to ~426 nm and the appearance of a characteristic α-peak at 560 nm and β-peak at 530 nm (Fig. 4G; Fig. S4). All variants, regardless of synthase function, showed similar UV-vis spectra, characteristic of this hemoprotein. Thus, the functional defects are likely not due to a defect in heme interaction. A caveat is that UV-vis spectral analysis provides an average of the heme environment, thus while the observed spectra of the variants appear to be substoichiometric compared to wildtype, this likely reflects differences due either to variable amounts of co-purification of holoCcmE or to transient interactions with CcmF P-heme (from holoCcmE), as the stoichiometric TM-*b-*heme (22) is anticipated to be similar.

### Cysteine/heme crosslinking reveals the CcmF WWD domain functions to bind heme

To more closely examine the role of the CcmF WWD domain in heme binding, the cysteine/heme crosslinking approach was utilized. This approach exploits the natural propensity of cysteine and heme to form a covalent bond when in close proximity (Fig. S5A), similar to heme attachment to the CXXCH motif of cytochrome *c* (4, 15, 16), thus can covalently trap (or crosslink) heme within a heme interaction domain (15, 16). The cysteine/heme crosslinking approach determined that heme directly interacts with residues in the WWD domains of CcmC (16) and CcsBA (15) (Fig. 2B, see the asterisks). Subsequent Cryo-EM structures of CcmABCD (18) and CcsBA (17) confirmed heme presence in the WWD domains of these proteins, further validating this approach to identify heme-handling domains.

**Fig 5:**
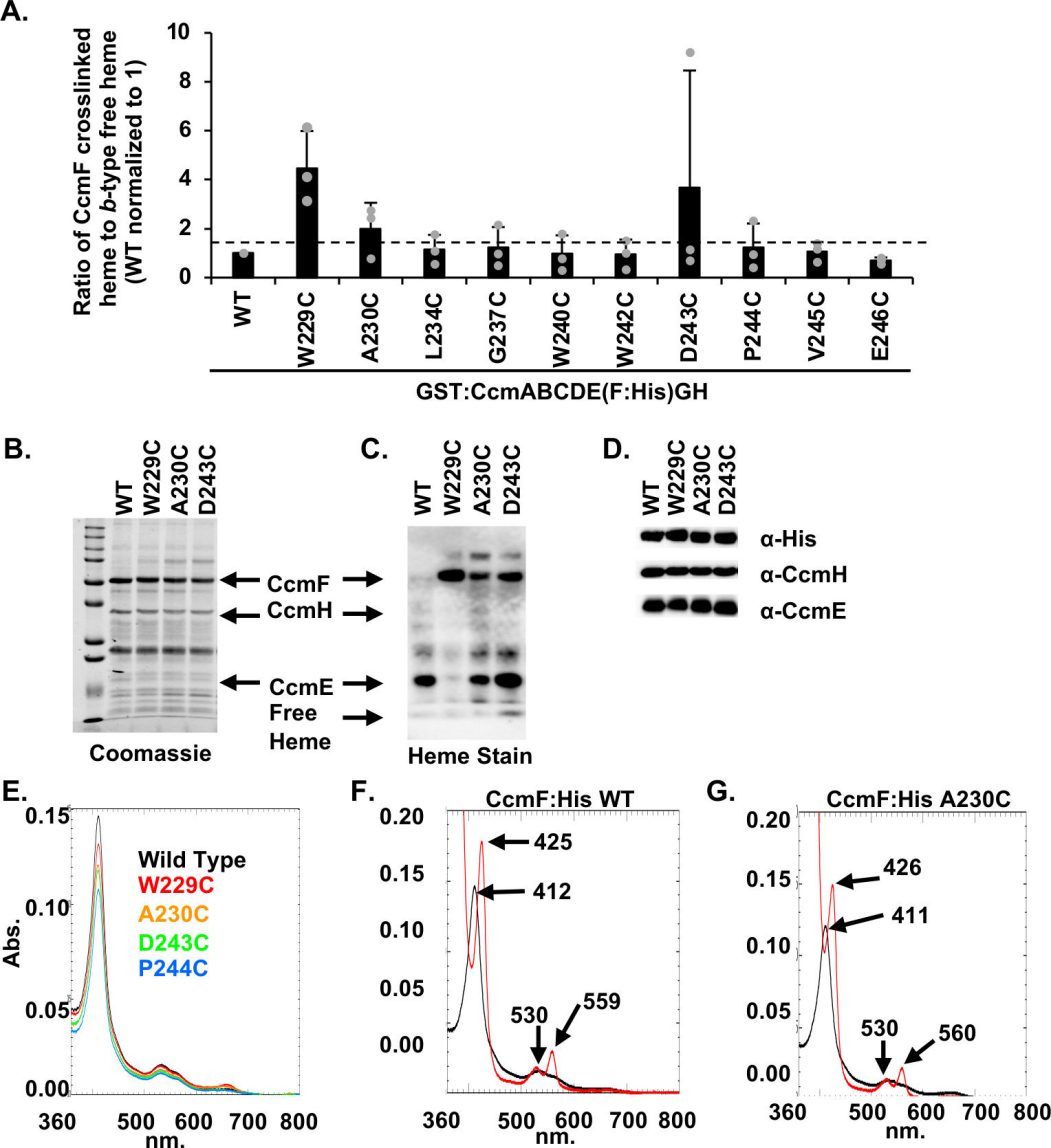
The CcmF WWD domain binds heme. Each residue of the CcmF:His wild-type or WWD domain single amino acid cysteine point mutations was engineered in the context of the full System I pathway (CcmA-H) plasmid. CcmF:His WWD cysteine variants were affinity purified and analyzed for the formation of a cysteine/heme crosslink by determining the ratio of CcmF-bound heme to *b*-type heme. Variants with a ratio of >1.5 or residues that had been shown to form a cysteine/heme crosslink in CcmC or CcsBA were chosen for further analysis. (**A**) CcmF to *b*-type heme quantitation from 10 key variants. Representative quantitation of three independent purifications. (**B and C**) Three cysteine variants formed the cysteine/heme crosslink. Five micrograms of affinity-purified protein was separated by SDS-PAGE and analyzed for protein stability (**B**) and formation of cysteine/heme crosslink via heme stain (**C**). (**D**) The CcmF:His WWD cysteine variants contain the 6×His tag and co-purify with CcmH and CcmE. (**E**) Heme co-purification was assayed via heme Soret (410 nm) with 50 µg of affinity-purified protein. Representative of three independent purifications. (**F and G**) UV-vis spectra of 50 µg of as-purified (black) and reduced (red) with key peaks indicated of WT and A230C variant. Representative of three independent purifications.

Twenty-two residues of the CcmF WWD domain (A228–S249) were individually mutated to cysteine on a plasmid containing the complete System I pathway [GST:CcmABCDE(F:6×His)GH]. CcmF WWD cysteine variants were affinity purified via a C-terminal 6×His tag and assessed for cysteine/heme crosslink formation via heme stain. Upon SDS-PAGE, crosslinked heme will be retained on the CcmF polypeptide, while *b*-type heme located in the transmembrane or WWD domain will dissociate from the protein polypeptide and run in the dye front as “free” heme. The ratio of CcmF bound heme to “free” or *b*-type heme was determined and variants with a ratio > 1.5 were selected. Note, due to the stably bound TM-*b-*heme associated with CcmF, increased free heme necessitated a lower ratio than prior analyses with CcmC and CcsBA, neither of which contain a stably bound *b*-heme in the protein. Of the twenty-two CcmF WWD cysteine variants, we identified 10 variants for further analysis (Fig. 5A; Fig. S5B and C). These variants either had a ratio of CcmF:free heme of >1.5 (W229C, A230C, D243C, and P244C) and/or were residues previously identified to crosslink in CcmC and/or CcsBA (W229C, L234C, W240C, W242C, D243C, V245C, and E246C) (see Fig. 2B).

Further analysis determined that CcmF WWD cysteine variants W229C, A230C, and D243C formed a cysteine/heme crosslink (Fig. 5C) based on heme retention at the CcmF polypeptide upon SDS-PAGE, as demonstrated in previous cysteine/heme crosslinking analyses (15, 16). Note that heme retention at the CcmF polypeptide was not seen in the alanine variants, thus is specific to cysteine (Fig. 4E). All cysteine variants were stable (Fig. 5B; Fig. S5B), co-purify with CcmH and CcmE (Fig. 5D; Fig. S5D, F, and H), and co-purify with heme, as determined by analysis of the UV-vis Soret peak (410 nm) (Fig. 5E; Fig. S6A). Note that 410 nm UV-vis spectral analysis (Fig. 5E) accounts for the total heme in each sample, including heme from co-purified holoCcmE, as well as P-heme and TM-*b*-heme in CcmF, while heme stain analysis specifically identifies crosslinked heme (Fig. 5C). UV-vis spectral analysis of reduced CcmF WWD cysteine variants showed the characteristic Soret shift from 410 to ~426 nm and appearance of characteristic α-peaks at ~560 nm and β-peaks at ~530 nm (Fig. 5F through G; Fig. S6B through J). Thus, similar to alanine variants, cysteine substitutions did not alter the overall heme environment in CcmF. Of interest, there is a faint high molecular weight heme staining band in a subset of CcmF WWD cysteine variants (Fig. 5C; Fig. S5C). Immunoblotting determined that this complex is composed of CcmF, CcmH, CcmE, and heme (Fig. S5D through I). However, the formation of this complex is not due to the cysteine/heme crosslink (Fig. S5D through F), as it is present in the majority of CcmF cysteine substitutions.

**Fig 6:**
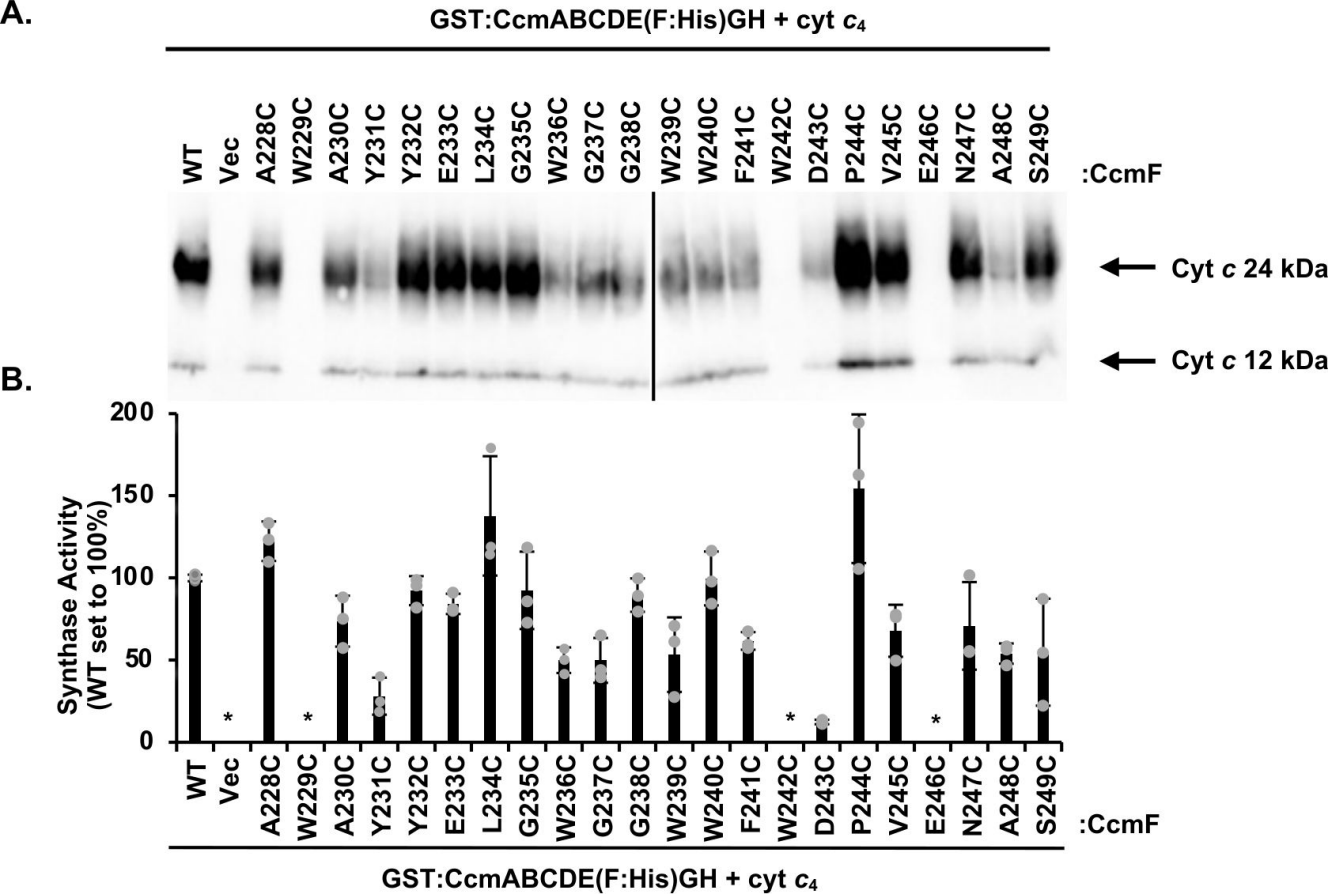
The WWD domain cysteine mutations impact CcmFH holocytochrome *c* synthase function. (**A**) CcmF:His wild-type or cysteine variants in the context of the complete System I pathway (CcmA-H) were recombinantly co-expressed with cytochrome *c*_4_:His in *E. coli* Δ*ccm* and synthase activity was determined as in Fig. 3. Three biological replicates, each containing three technical replicates, were performed. Representative samples are shown. (**B**) Quantitation of the CcmF:His wild-type and cysteine variant synthase function. WT is normalized to 100% function. Error bars show the standard deviation from the mean, and dots indicate individual data points. Representative biological replicate is shown. The asterisks indicate below detectable limit of heme stain.

To determine if the CcmF WWD cysteine variants impacted CcmF synthase function, the variants were recombinantly co-expressed with cytochrome *c*_4_ in *E. coli* Δ*ccm*. Three cysteine substitutions were defective for synthase function (W229C, W242C, and E246C) and three variants had severely reduced synthase function (<30%) (Y231C, G237C, and D243C). Ten variants retained partial synthase function (30%–80%) (A230C, E233C, G235C, W236C, W239C, F241C, V245C, N247C, A248C, and S249C) and six variants had wild-type function (>80%) (A228C, Y232C, L234C, G238C, W240C, and P244C) (Fig. 6; Fig. S7). Interestingly, two of the three cysteine/heme crosslinking variants were non-functional (W229C) or had severely impaired function (D243C), while one cysteine/heme crosslinking variant retained partial function (A230C).

### Conservation of heme binding in the heme-handling WWD protein family

**Fig 7:**
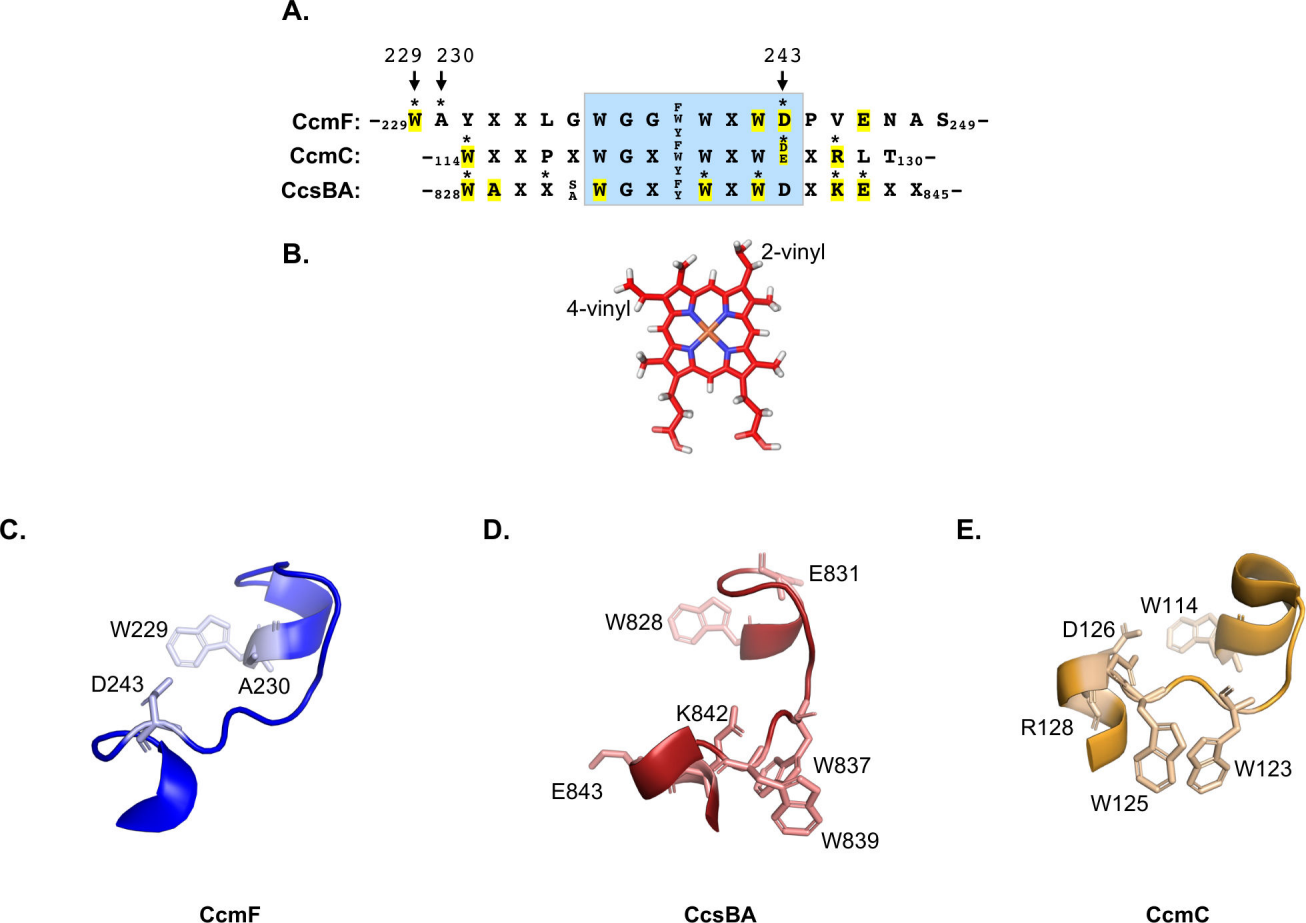
Comparison of WWD heme interactions’ domains. The WWD domains of CcmF, CcsBA, and CcmC are compared. (**A**) Sequence alignment of the WWD domains. Residues that form cysteine/heme crosslinks are denoted with an asterisk, and CcmF residues are indicated by amino acid number. Residues that are important for cytochrome *c* biogenesis, as determined by the reduction of cytochrome *c* biogenesis by >75% when mutated to cysteine are highlighted in yellow (see Fig. 6) (15, 16). (**B**) Structure of heme with 2 and 4-vinyl groups labeled, Fe is shown in orange. Heme is oriented based on its known stereospecific positioning within the CcmC (16, 18) and CcsBA (15, 17) WWD domains, where the 4-vinyl is positioned near N-terminus of the domain and the 2-vinyl near the C-terminus. (**C–E**) The structure of the WWD domains of the cytochrome *c* biogenesis heme-handling proteins is shown with cysteine/heme crosslinking residues indicated (**C**) CcmF (blue, PDB: 6ZMQ). Note, *T. thermophilus* structure is shown, no heme in the WWD domain, labeled with *E. coli* residue numbers to correspond with Fig. 5. (**D**) CcsBA [red, “open” structure with heme (not shown) in the WWD domain PDB: 7S9Y], and (**E**) CcmC (gold, “closed” structure with heme [not shown] in the WWD domain PDB: 7F04).

Structure-function analysis of all WWD domains of the HHP family has now been undertaken and residues that directly interact with heme have been identified via cysteine/heme crosslinking in CcmF (Fig. 5), CcmC (16), and CcsBA (15) (Fig. 7A, C, and D). Despite the high sequence conservation within the WWD domains, the specific residues that interact with heme differ (Fig. 7A, asterisks). However, in all cases, there are crosslinking residues located at the N or C-termini of the WWD domain, likely indicating a similar mechanism of heme handling and potential heme stereospecificity across this protein family. In CcmC, stereospecific positioning of heme with the 4-vinyl located near N-terminal W114 and the 2-vinyl of heme located near C-terminal D126/R128 was determined (16, 18). Similarly, the cryo-EM structure of CcsBA revealed the 4-vinyl of heme near the N-terminal W828 residue and the 2-vinyl near the C-terminal W839 residue (17). AF2Complex structural modeling of CcmFH predicts that CcmF residue W229 is in proximity to 4-vinyl of P-heme (37), while confirmation of heme orientation awaits structural determination, this interaction is demonstrated by cysteine/heme crosslinking (Fig. 5C). Thus, heme is stereospecifically positioned in all WWD domains of the bacterial cytochrome *c* biogenesis pathways (Fig. 7). However, CcmF lacks heme interaction residues in the central area of the WWD domain, a difference from CcmC and CcsBA. This may be indicative of differences in heme delivery to the WWD domains.

## DISCUSSION

A comprehensive structure-function analysis of the CcmF WWD domain has been undertaken utilizing both alanine scanning and cysteine/heme crosslinking to identify residues that are critical for CcmF synthase function. We report the first evidence that CcmF directly interacts with P-heme in the WWD domain (Fig. 5A and C) and identify WWD residues absolutely required for CcmF synthase function (Fig. 3 and 6). The WWD domain is neither required for CcmF interaction with CcmH (Fig. 4C and 5D) and CcmE (Fig. 4D and 5D) nor does it have a significant impact on overall levels of heme co-purification or heme environment within affinity-purified CcmF (Fig. 4F, G, and 5E through G; Fig. S4 and S6). The lack of impact on heme co-purification and heme environment is unsurprising given that the stable transmembrane TM-*b-*heme is predicted to be in a 1:1 stoichiometry with CcmF (22), thus likely masks any potential quantitative differences in the more transient WWD P-heme.

Of interest, alanine and cysteine mutational studies demonstrated that the WWD domain is required for CcmF’s role in heme attachment to apocytochrome *c* (i.e., synthase function), specifically CcmF WWD residues W229 and E246 are absolutely required for heme attachment under both alanine and cysteine substitution analyses (Fig. 3 and 6). Thus, holocytochrome *c* synthase phenotypes are likely not due to a conformational change in the domain due to the single amino acid point mutation, but rather due to loss of the residue. These results reveal similarity to the System II holocytochrome *c* synthase, CcsBA, which required homologous residues W828 and E843 (compared to CcmF W229 and E246) for synthase function (see Fig. 7B) (15). Interestingly, CcsBA also required W833 (compared to CcmF W236) for synthase function (15), while CcmF does not, suggesting mechanistic differences may exist between the bacterial holocytochrome *c* synthases. It is perhaps surprising that only two variants were completely deficient given that over 10 residues of the CcmF WWD domain are completely conserved. We suggest that there is a built-in redundancy for binding heme and that single substitutions retain function, yet the entire domain has evolved to optimize interactions with heme.

Cysteine/heme crosslinking analysis identified three CcmF WWD residues W229, A230, and D243 that when mutated to cysteine directly interact with P-heme as demonstrated by the formation of a cysteine/heme crosslink (Fig. 5C). Thus, these residues are essential for P-heme interaction in the native protein and provide the first direct evidence of P-heme localization in the CcmF WWD domain. Of note, CcmF A230C had lower levels of crosslinked heme (Fig. 5C) in cysteine/heme crosslinking assays. In functional studies of the CcmF WWD cysteine variants (Fig. 6), where apocytochrome *c* is present, A230C retains function, suggesting heme attachment to the CXXCH motif is favored over the formation of the cysteine/heme crosslink. Alternatively, A230C’s lower levels of crosslinked heme could result in a heterogenous protein population consisting of A230C with and without crosslinked heme, accounting for the observed synthase activity. In contrast, W229C and D243C displayed higher levels of crosslinked heme and in functional assays had no or severely reduced synthase activity, respectively. These results suggest that these residues display a stronger interaction with P-heme. Of interest, a recent study of structural predictions utilizing AF2Complex analyzed the System I pathway, including predictions of heme localization in the CcmF WWD domain and identified W229 as a heme interacting residue (37), which our experimental results support.

All CcmF cysteine/heme crosslinks occur near the terminal ends of the WWD domain, located close to the transmembrane alpha-helices (Fig. 7C). Both CcmC (16) (Fig. 7E) and CcsBA (17) (Fig. 7D) had similar requirements for heme interaction via residues located at the ends of the WWD domain, suggesting the orientation of heme within this domain is likely highly conserved (see Fig. 7B). Of note, both CcmC and CcsBA contain additional residues that interact with heme on the central loop of the WWD domain (Fig. 7A, D, and E) (15–18), indicating requirements for WWD heme localization may differ across the HHP family. Based on previous studies, it is known that P-His1 and P-His2 are required for CcmF holocytochrome *c* synthase function and act as heme ligands (22, 26, 31). Similarly, P-His1 and P-His2 are required for CcmC and CcsBA heme attachment function and ligand the WWD localized heme (15, 17, 18, 23, 41). Therefore, the active site for CcmF holocytochrome *c* synthase activity and P-heme interaction domain consists of the WWD domain with P-heme ligated by P-His1 and P-His2.

### Model of CcmF function

Based on this structure-function analysis, a model for CcmFH P-heme interaction and holocytochrome *c* synthase function is proposed in which CcmFH receives the WWD domain P-heme from holoCcmE (4, 24, 27, 28, 43). Recent structural analysis of CcmF suggests that CcmE may transfer heme to the CcmF WWD domain via the outer leaflet of the membrane (32); however, the mechanism of heme delivery awaits further experimental evidence. In the CcmF WWD domain, P-heme is stereospecifically positioned for attachment to the apocytochrome *c* CXXCH motif via WWD residues W229, A230, and D243 and with axial ligands P-His1 and P-His2 (31). For apocytochrome *c* heme attachment to occur, heme and the CXXCH thiols must be in a reduced state. The CcmF TM-*b-*heme is proposed to play a role in the reduction of the WWD P-heme (22), while CcmG and CcmH function in the reduction of the CXXCH cysteine thiols (44–48). CcmH is known to interact with apocytochrome *c* and likely plays a role in the positioning of the CXXCH for heme attachment (37, 47, 49–52). Prior to heme attachment, ligand switching from P-His2 to the His of CXXCH is proposed to occur. The recent crystal structure of CcmF indicates that P-His2 is on a flexible loop with less density, suggesting it may undergo a conformational change (32) and is likely responsible for ligand switching. Next, heme is covalently attached to the cytochrome *c* CXXCH motif (26), followed by the release of holocytochrome *c*. The molecular details of heme attachment and release await additional experimental evidence.

### Comparison of heme handling protein family

This study facilitates a comparison of the HHP family (38) WWD domains from CcmF (System I), CcmC (System I), and CcsBA (System II) (Fig. 7; Table 1). Despite encoding the conserved WWD domain, each HHP protein functions for a different purpose in cytochrome *c* biogenesis, requires different protein-protein interactions to function, and are predicted to receive heme in the WWD domain via unique mechanisms (see Table 1). Yet, all three proteins have now been shown to directly interact with heme in the WWD domain via cysteine/heme crosslinking (Fig. 5) (15, 16). Heme is stereospecifically positioned in the WWD domain via residues near the alpha-helices, liganded by P-His1/P-His2 (15, 17, 18, 22, 23, 25, 31, 41), and recent structural determinations of each HHP suggest that P-His2 is located on a flexible loop that likely undergoes conformational changes to initiate ligand switching for heme transfer (17, 18, 32) (see Table 1). Thus, a direct comparison of these WWD domains in the context of their protein functions provides important initial insights into global mechanisms of heme interaction domains.

**TABLE 1:** Comparison of the heme-handling protein family

	Heme-handling protein		
	CcmC	CcmF	CcsBA
Function	Required for heme attachment to CcmE	Holocytochrome *c* synthase	Holocytochrome *c* synthase
	Component of CcmABCD ABC transporter release complex		Putative heme transporter
Protein partner required for function	CcmD	CcmH	n/a^*a*^
Heme attachment to	CcmE	Apocytochrome *c*	Apocytochrome *c*
Heme interaction domain/axial ligands	WWD domain/P-His1, P-His2	1:1 TMD *b*-heme/TM-His1, TM-His2	TMD/TM-His1, TM-His2
		WWD domain/P-His1, P-His2	WWD domain/P-His1, P-His2
Putative active site	WWD + P-His1, P-His2	WWD + P-His1, P-His2	WWD + P-His1, P-His2
Heme delivery to the WWD domain	Unknown	holoCcmE	Proposed TMD → WWD

^*a*^ n/a = not applicable.

## MATERIALS AND METHODS

### Bacterial growth conditions

*Escherichia coli* strains were grown in Luria-Bertani broth (LB; Difco) at 37°C at 200 rpm with appropriate selective antibiotics (carbenicillin, 50 µg/mL; chloramphenicol, 20 µg/mL) and/or inducing reagents (isopropyl-D-1-thiogalactopyranoside [IPTG; GoldBio], 1.0 or 0.1 mM; L-arabinose [GoldBio], 0.2% [wt/vol]).

### Construction of alanine and cysteine variants

All cloning was performed in *E. coli* NEB-5α. Single amino acid alanine and cysteine substitutions were engineered using the QuikChange II site-directed mutagenesis kit (Agilent Technologies) and verified by DNA sequencing. A complete list of strains, plasmids, oligonucleotide primers, and templates is provided in Table S1.

### CcmF structural modeling

The predicted *E. coli* (K12) CcmF (UniProt: P33927) was obtained using AlphaFold DB version 2022-06-01 under the AlphaFold v2.0 pipeline (35, 36). Most of the predicted structures indicated a high level of model confidence (per residue confidence score, pLDDT, greater than 90, https://alphafold.ebi.ac.uk/entry/P33927). The PDB files of *E. coli* and *T. thermophilus* CcmFs (PDB: 6ZMQ) (32) were uploaded and compared using PyMOL (version 2.5.2).

### *In vivo* cytochrome *c* biogenesis assays

The CcmF variants were expressed in the context of the full System I pathway and co-expressed with cytochrome *c*_4_:His (pRGK332) in RK103 as previously described (26, 53). Starter cultures were back diluted 1:5 into 5 mL of LB with appropriate antibiotics and grown for 3 hours at 37°C and 200 rpm. Proteins were induced with 0.1 mM IPTG and 0.2% arabinose and grown for 3 hours at 37°C and 200 rpm. Cells were collected by centrifugation and frozen at −80°C. Two hundred microliters of Bacterial Protein Extraction Reagent (B-PER, Thermo Scientific) was used to lyse cells per the manufacturer’s instructions. A total of 50 µg of total cell lysate was separated by SDS-PAGE and cytochrome *c* biogenesis (i.e., heme attachment) was monitored by heme stain.

### Heme stains, immunoblots, and quantification

Heme staining was performed as previously described utilizing an ECL-based development and CCD imaging (15, 16, 54). Immunoblots were performed on 5 µg of affinity-purified protein and probed with the following antibodies: α -His-HRP (1:90,000) (Sigma-Aldrich A7058), α -CcmE (1:7,500) (21), or α -CcmH (1:30,000) (21). Protein A peroxidase (Millipore Sigma, P8651) was used as a secondary label as needed. Imaging was performed with Azure Sapphire Bimolecular Imager (Azure, SPC11-0239) and quantified with AzureSpot Software (Azure, version 1.3).

### Protein affinity purifications

Affinity purifications of C-terminal CcmF:6×His were performed as previously described (26, 43) with minor modifications. RK103 *E. coli* ∆*ccm* (53) was used for protein expression.

### UV-visible absorption spectroscopy

UV-visible absorption spectra were collected with a UV-1900i and LabSolutions software (Shimadzu; LabSolutions UV-Vis version 1.10) and performed as described in reference 26 with the following modifications: 50 µg of protein in the buffer used for purification was used to collect spectra and to obtain quantitation of total heme levels using the Soret region. Sodium hydrosulfite powder (Sigma 157-953) was used to reduce protein spectra.

## SUPPLEMENTAL MATERIAL

Fig. S1 mbio.01509-23-s0001.tif Alpha-fold predicted structure of *E. coli* CcmF.

Fig. S2 mbio.01509-23-s0002.tif The WWD “core regions” of CcmF, CcsBA, and CcmC.

Fig. S3 mbio.01509-23-s0003.tif The WWD domain is required for CcmFH synthase function.

Fig. S4 mbio.01509-23-s0004.tif CcmF WWD alanine mutations do not disrupt heme environment.

Fig. S5 mbio.01509-23-s0005.tif Cysteine/heme cross-linking in the CcmF WWD domain.

Fig. S6 mbio.01509-23-s0006.tif UV-vis spectral analysis of CcmF reveals the stable TM-b-heme is not impacted by WWD cysteine variants.

Fig. S7 mbio.01509-23-s0007.tif Function of the CcmF WWD cysteine variants.

Table S1 mbio.01509-23-s0008.pdf Strains, plasmids, and primers employed in this study.

ASM does not own the copyrights to Supplemental Material that may be linked to, or accessed through, an article. The authors have granted ASM a non-exclusive, world-wide license to publish the Supplemental Material files. Please contact the corresponding author directly for reuse.
