# Supplementary figures and images for: Structure-function analysis of the heme-binding WWD domain in the bacterial holocytochrome c synthase, CcmFH
Source: mBio. 2023 Nov 6;14(6):e01509-23. doi: 10.1128/mbio.01509-23 (PMC10746174; doi:10.1128/mbio.01509-23)

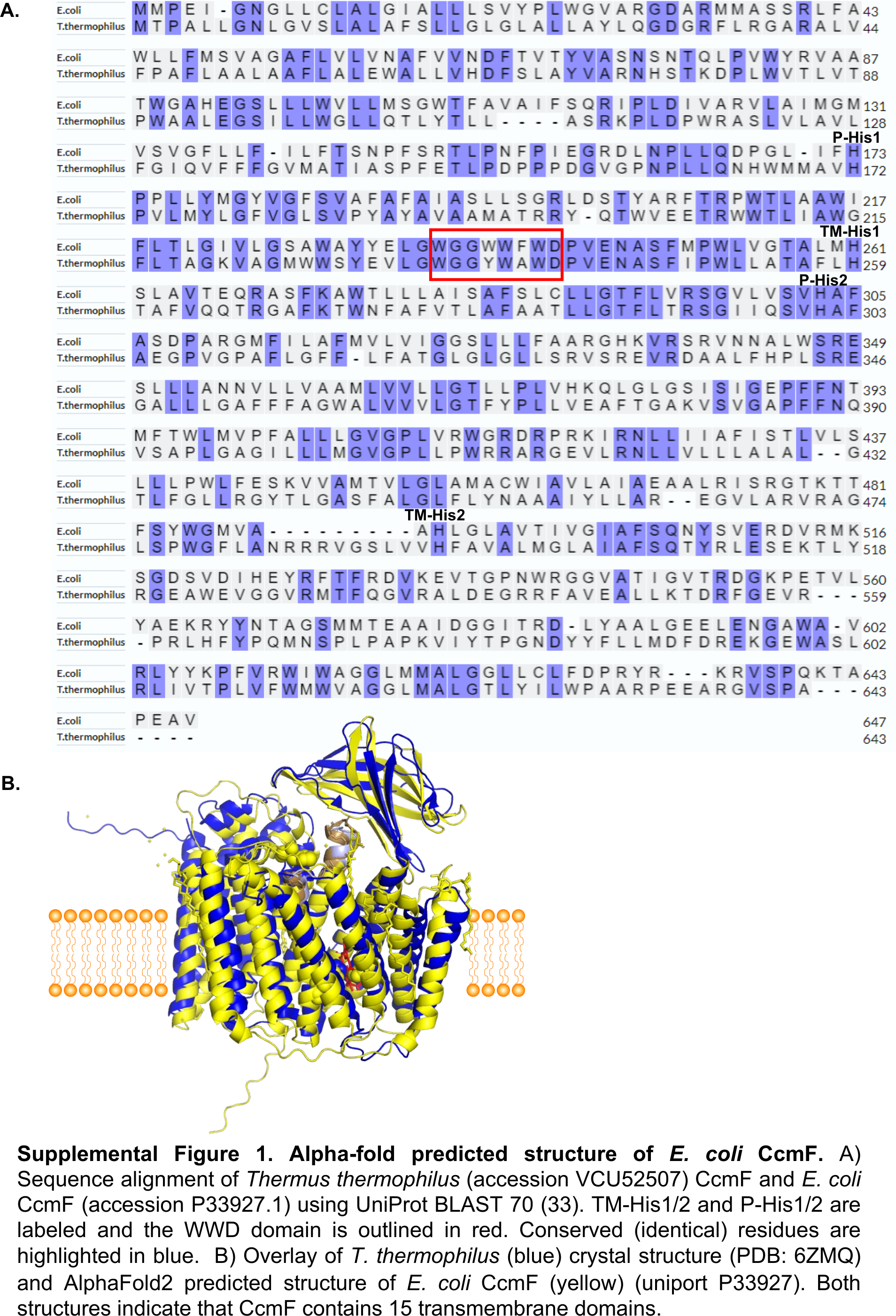

Supplement: Fig. S1 — Alpha-fold predicted structure of E. coli CcmF. [file mbio.01509-23-s0001.tif]

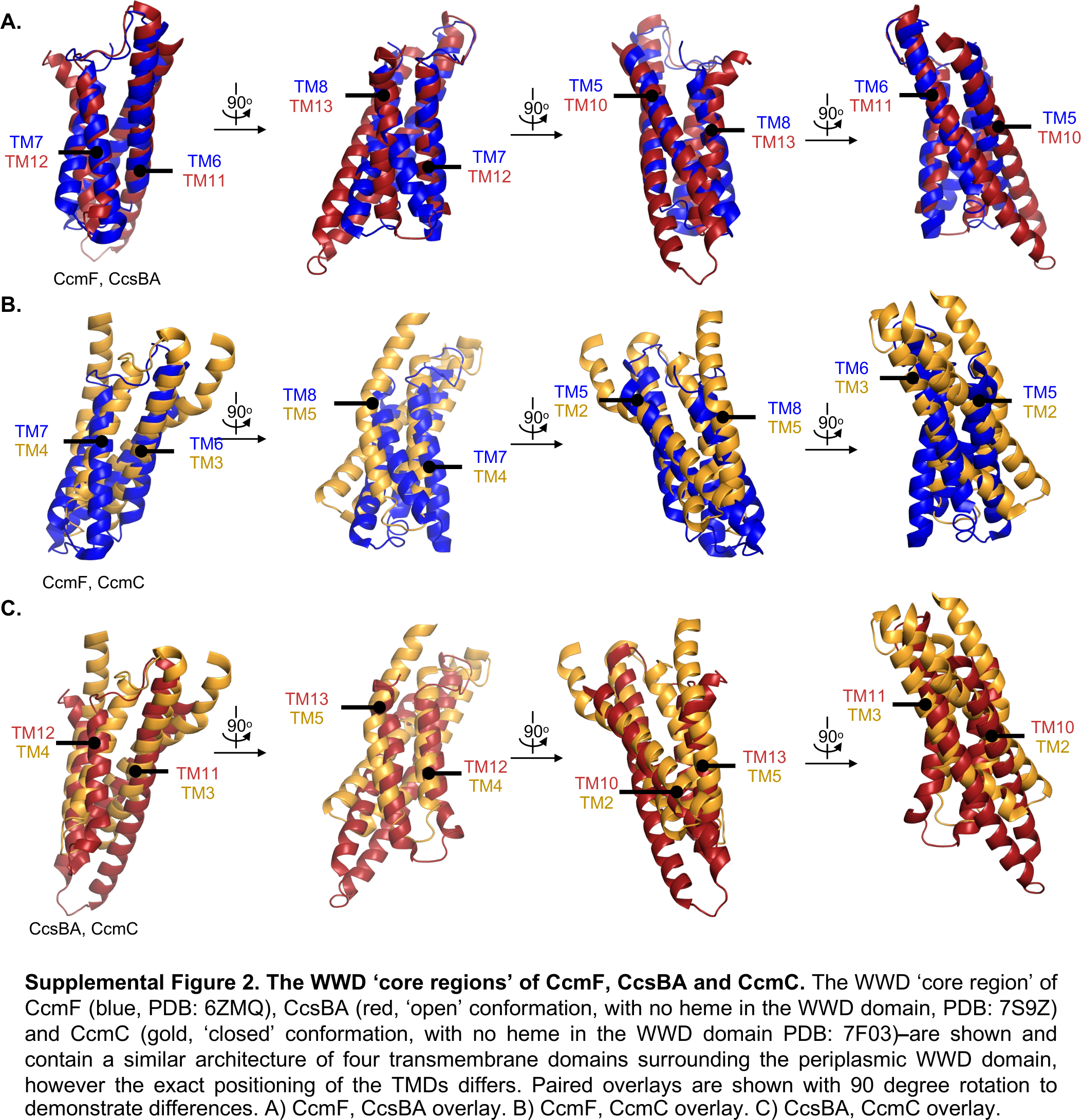

Supplement: Fig. S2 — The WWD “core regions” of CcmF, CcsBA, and CcmC. [file mbio.01509-23-s0002.tif]

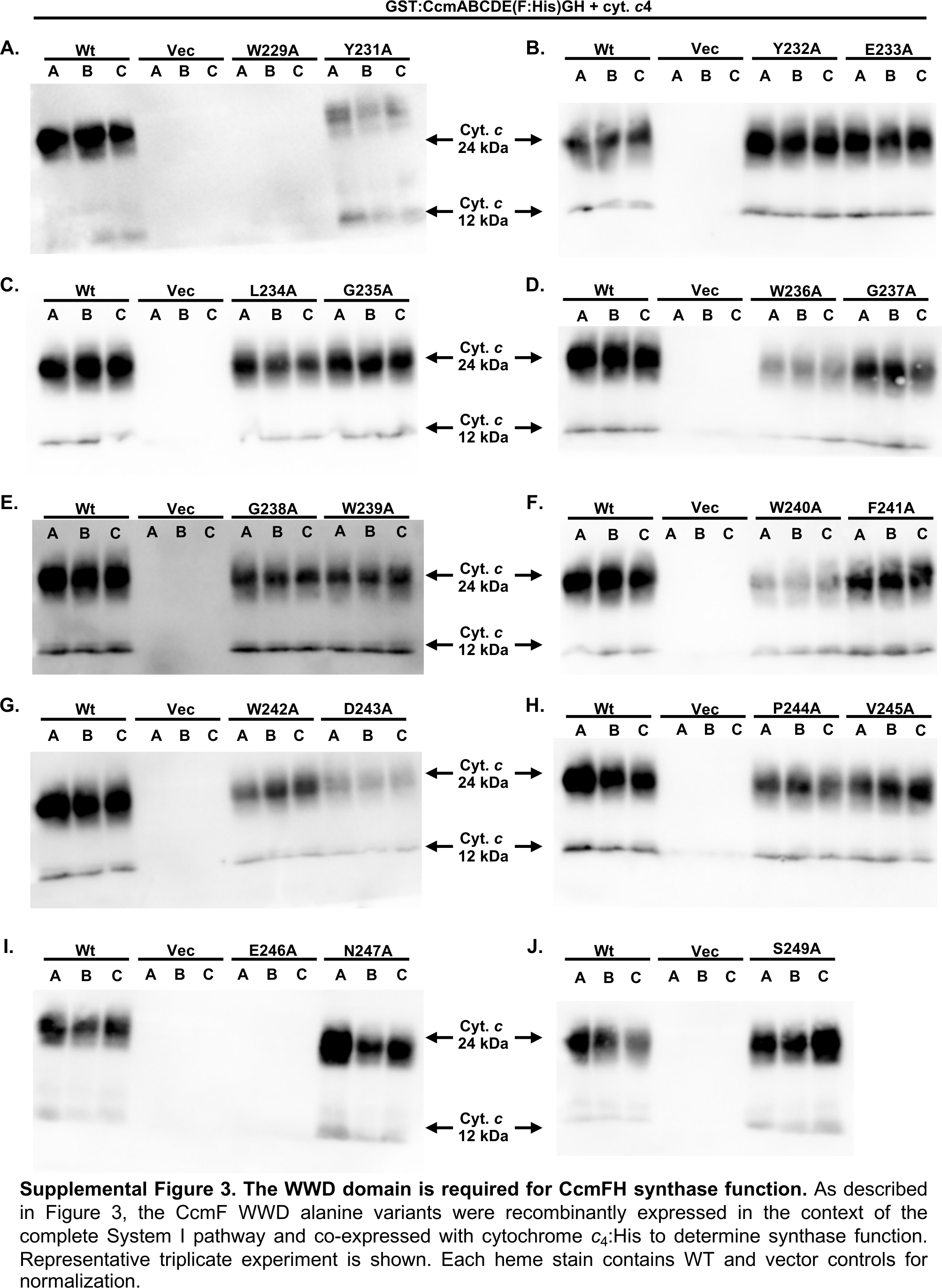

Supplement: Fig. S3 — The WWD domain is required for CcmFH synthase function. [file mbio.01509-23-s0003.tif]

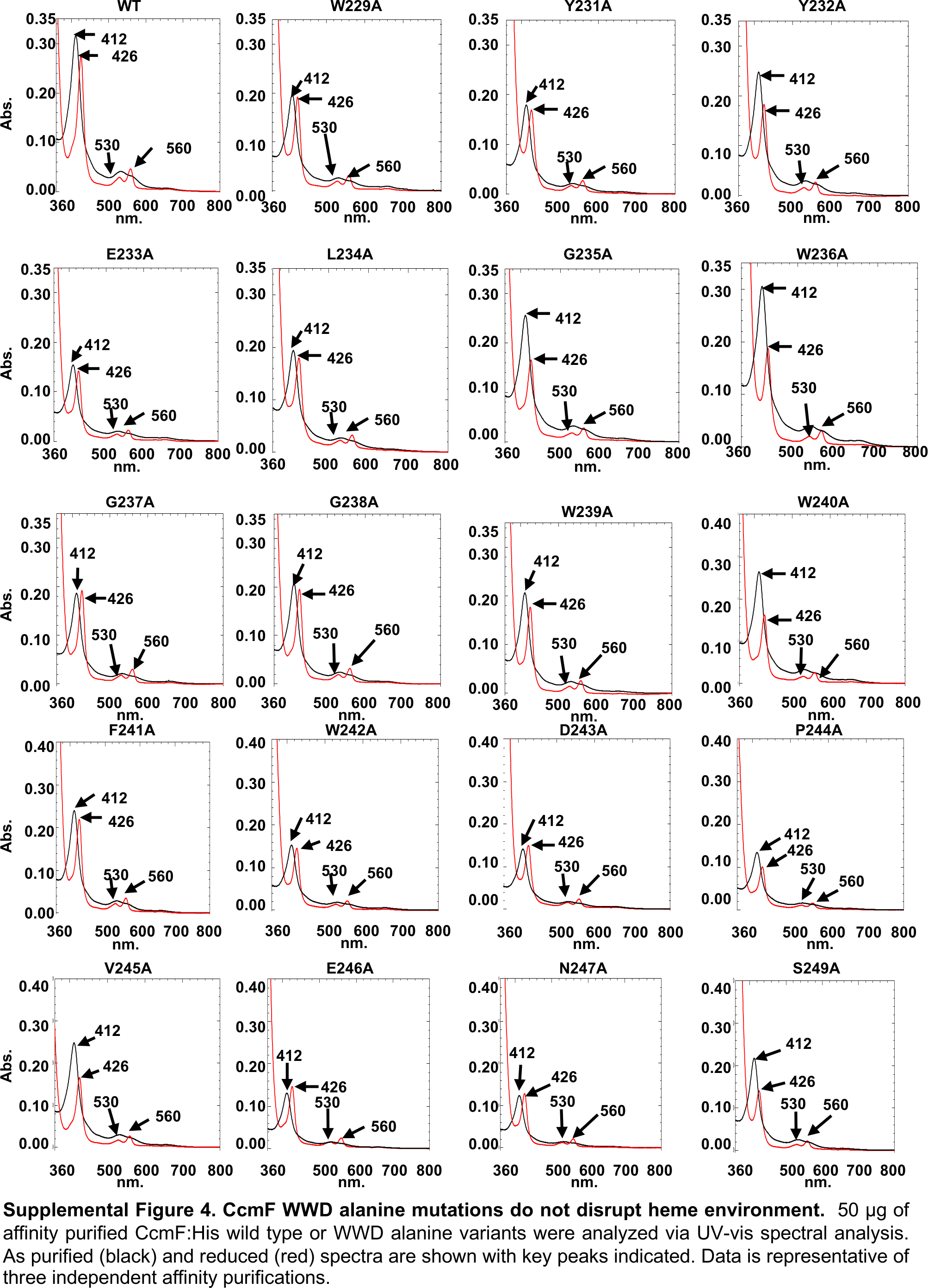

Supplement: Fig. S4 — CcmF WWD alanine mutations do not disrupt heme environment. [file mbio.01509-23-s0004.tif]

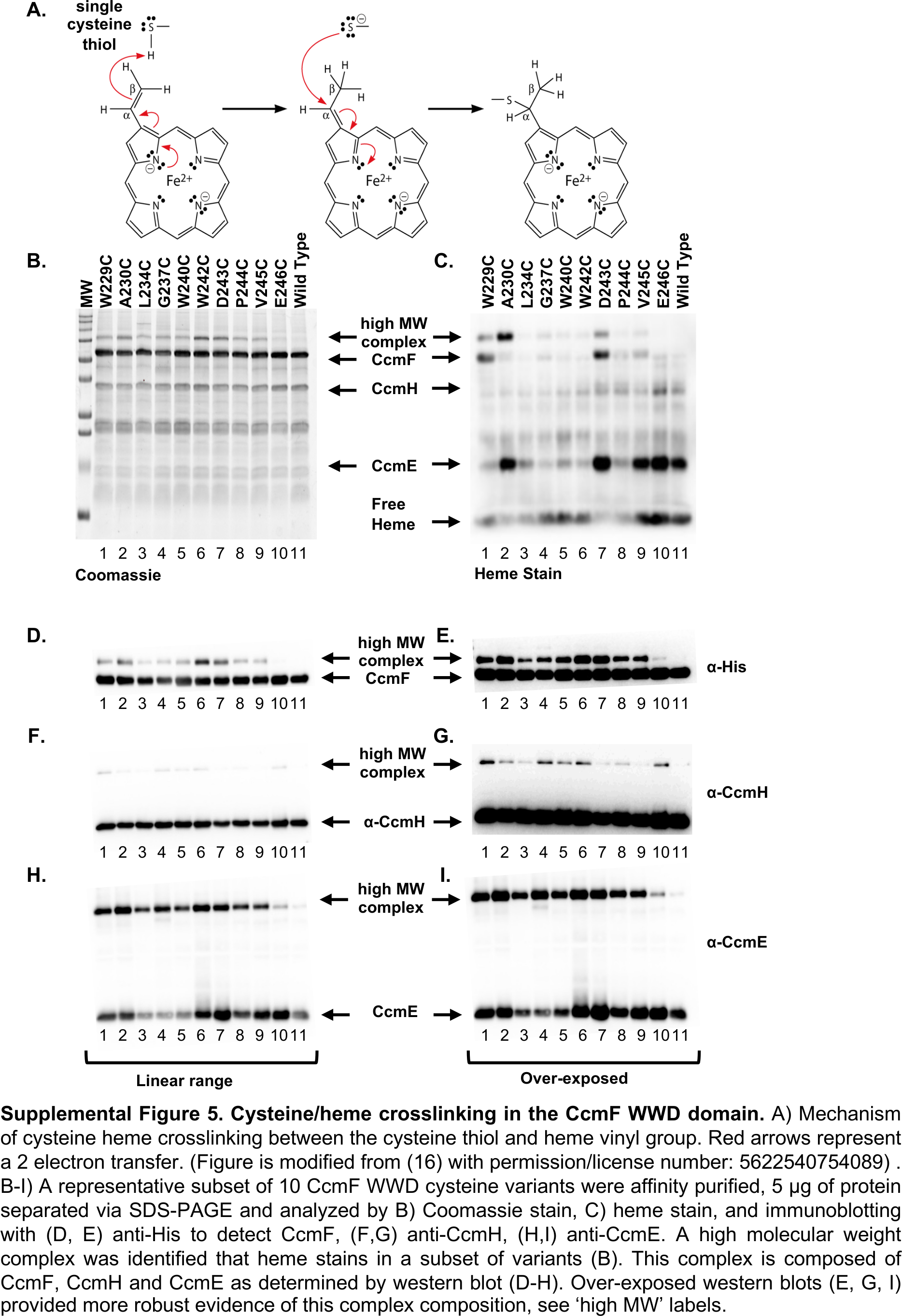

Supplement: Fig. S5 — Cysteine/heme cross-linking in the CcmF WWD domain. [file mbio.01509-23-s0005.tif]

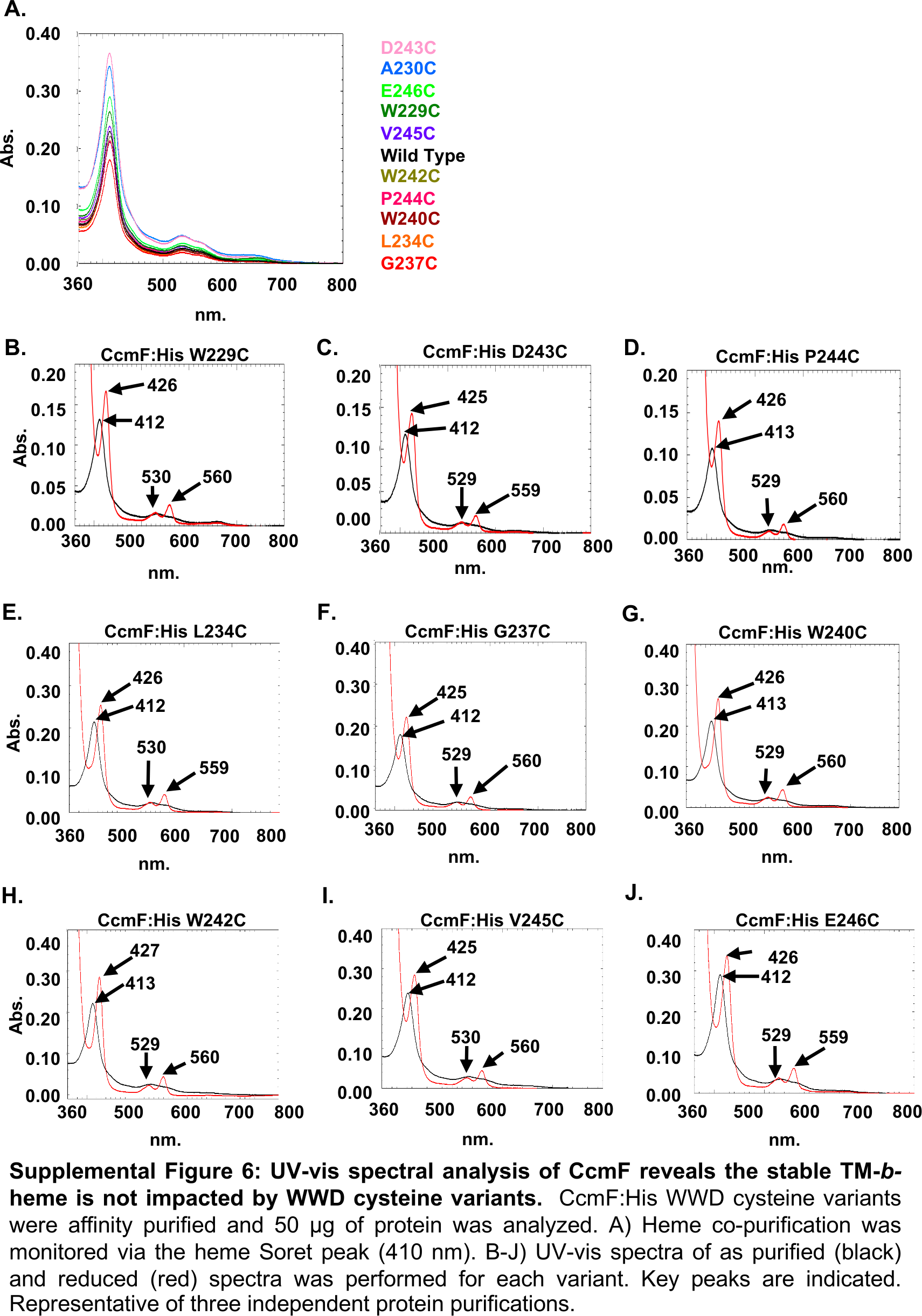

Supplement: Fig. S6 — UV-vis spectral analysis of CcmF reveals the stable TM-b-heme is not impacted by WWD cysteine variants. [file mbio.01509-23-s0006.tif]

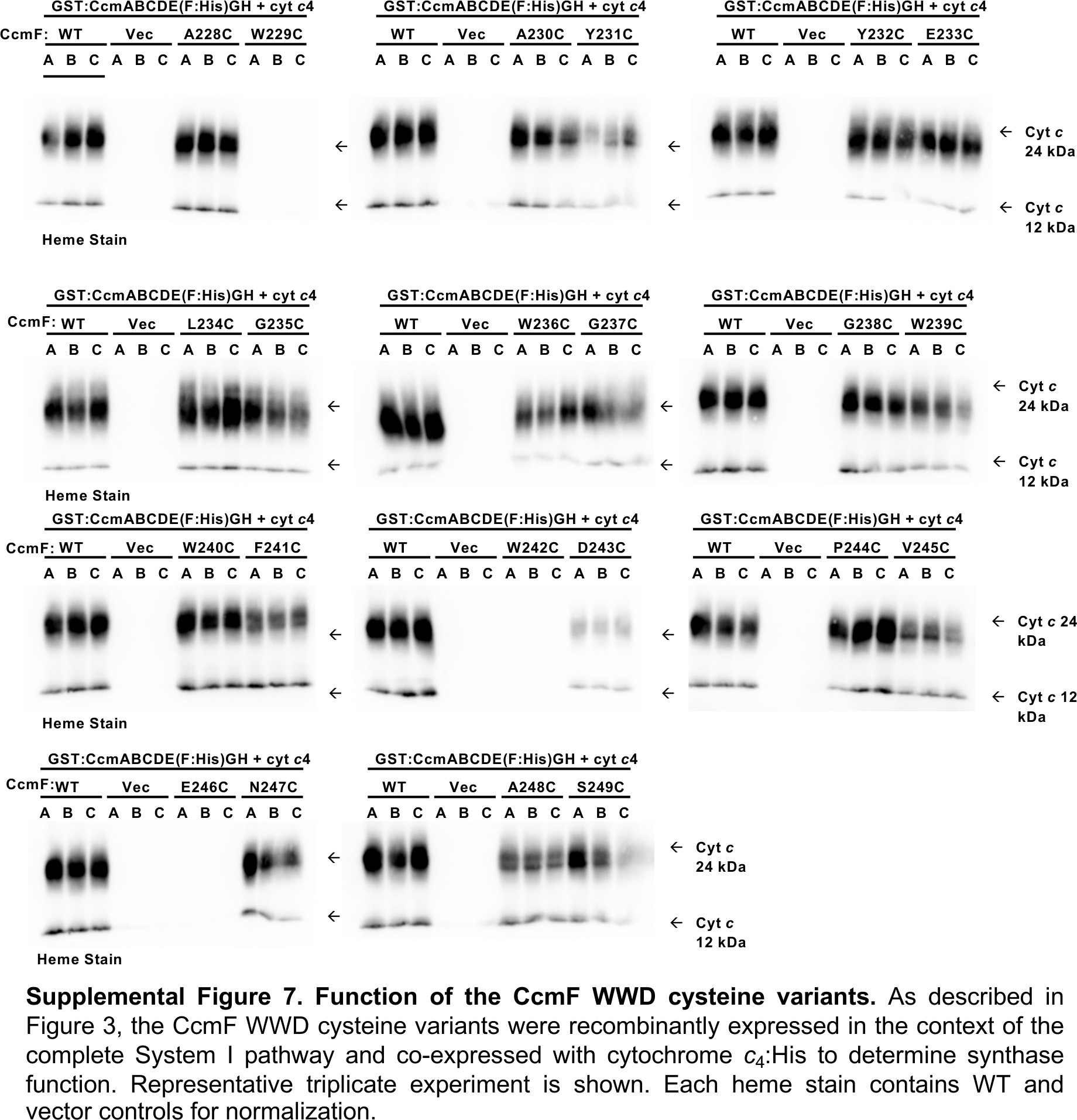

Supplement: Fig. S7 — Function of the CcmF WWD cysteine variants. [file mbio.01509-23-s0007.tif]
