# Supplementary material for: Structure-function analysis of the heme-binding WWD domain in the bacterial holocytochrome c synthase, CcmFH
Source: mBio. 2023 Nov 6;14(6):e01509-23. doi: 10.1128/mbio.01509-23 (PMC10746174; doi:10.1128/mbio.01509-23)
Supplement: Table S1 — Strains, plasmids, and primers employed in this study. [file mbio.01509-23-s0008.pdf]

Supplemental Table 1. Relevant Strains, Plasmids and Primers Employed in this Study

Strain, Plasmid	Description	Reference
E. coli		
RK103	*E. coli* MG1655 Δ*ccm::kan*^{R}, deleted for all *ccm* genes	*Feissner et al, 2006*
NEB 5-α	fhuA2 Δ(argF-lacZ)U169 phoA glnV44 Φ80 Δ(lacZ)M15 gyrA96 recA1 relA1 endA1 thi-1 hsdR17	
Plasmid		
pRGK332	pBAD cytochrome c4:His	*Feissner et al, 2006*
pRGK386	pGEX GST:CcmABCDE(F:His)GH	*Richard-Fogal, 2009*
pMCS721	pGEX GST:CcmABCDE(F:His)GH CcmF (V245A)	This Study
pMCS722	pGEX GST:CcmABCDE(F:His)GH CcmF (Y231A)	This Study
pMCS724	pGEX GST:CcmABCDE(F:His)GH CcmF (E246A)	This Study
pMCS725	pGEX GST:CcmABCDE(F:His)GH CcmF (W242A)	This Study
pMCS726	pGEX GST:CcmABCDE(F:His)GH CcmF (W236A)	This Study
pMCS728	pGEX GST:CcmABCDE(F:His)GH CcmF (G237A)	This Study
pMCS729	pGEX GST:CcmABCDE(F:His)GH CcmF (G238A)	This Study
pMCS730	pGEX GST:CcmABCDE(F:His)GH CcmF (W239A)	This Study
pMCS731	pGEX GST:CcmABCDE(F:His)GH CcmF (F241A)	This Study
pMCS732	pGEX GST:CcmABCDE(F:His)GH CcmF (P244A)	This Study
pMCS733	pGEX GST:CcmABCDE(F:His)GH CcmF (S249A)	This Study
pMCS734	pGEX GST:CcmABCDE(F:His)GH CcmF (W240A)	This Study
pMCS736	pGEX GST:CcmABCDE(F:His)GH CcmF (W229A)	This Study
pMCS738	pGEX GST:CcmABCDE(F:His)GH CcmF (L234A)	This Study
pMCS739	pGEX GST:CcmABCDE(F:His)GH CcmF (D243A)	This Study
pMCS740	pGEX GST:CcmABCDE(F:His)GH CcmF (Y232A)	This Study
pMCS741	pGEX GST:CcmABCDE(F:His)GH CcmF (G235A)	This Study
pMCS742	pGEX GST:CcmABCDE(F:His)GH CcmF (N247A)	This Study
pMCS744	pGEX GST:CcmABCDE(F:His)GH CcmF (E233A)	This Study
pMCS767	pGEX GST:CcmABCDE(F:His)GH CcmF (W229C)	This Study
pMCS769	pGEX GST:CcmABCDE(F:His)GH CcmF (A230C)	This Study
pMCS770	pGEX GST:CcmABCDE(F:His)GH CcmF (Y231C)	This Study
pMCS772	pGEX GST:CcmABCDE(F:His)GH CcmF (Y232C)	This Study
pMCS773	pGEX GST:CcmABCDE(F:His)GH CcmF (E233C)	This Study
pMCS774	pGEX GST:CcmABCDE(F:His)GH CcmF (L234C)	This Study
pMCS775	pGEX GST:CcmABCDE(F:His)GH CcmF (G235C)	This Study
pMCS777	pGEX GST:CcmABCDE(F:His)GH CcmF (W236C)	This Study
pMCS778	pGEX GST:CcmABCDE(F:His)GH CcmF (G238C)	This Study
pMCS916	pGEX GST:CcmABCDE(F:His)GH CcmF (W239C)	This Study
pMCS917	pGEX GST:CcmABCDE(F:His)GH CcmF (F241C)	This Study
pMCS918	pGEX GST:CcmABCDE(F:His)GH CcmF (D243C)	This Study
pMCS919	pGEX GST:CcmABCDE(F:His)GH CcmF (P244C)	This Study
pMCS920	pGEX GST:CcmABCDE(F:His)GH CcmF (V245C)	This Study
pMCS921	pGEX GST:CcmABCDE(F:His)GH CcmF (E246C)	This Study
pMCS922	pGEX GST:CcmABCDE(F:His)GH CcmF (N247C)	This Study

pMCS924	pGEX GST: CcmABCDE(F:His)GH CcmF (W240C)	This Study
pMCS925	pGEX GST: CcmABCDE(F:His)GH CcmF (G237C)	This Study
pMCS926	pGEX GST: CcmABCDE(F:His)GH CcmF (W242C)	This Study
pMCS927	pGEX GST:CcmABDE(F:His)GH CcmF (A248C)	This Study
pMCS929	pGEX GST:CcmABDE(F:His)GH CcmF (S249C)	This Study
pMCS1070	pGEX GST:CcmABCDE(F:His)GH CcmF (A228C)	This Study

Oligonucleotide	Sequence (5' --> 3')	Purpose	Template
MSP360	gcgcataaacgaggcgtttgctaccggatcccagaacc	pMCS724 cloning	pRGK386
MSP361	ggttctgggatccggtagcaaacgcctcgtttatgccg	pMCS724 cloning	pRGK386
MSP362	cgttttctaccggatccgcgaaccaccagccaccc	pMCS725 cloning	pRGK386
MSP363	gggtggctggtggttcgcggatccggtagaaaacg	pMCS725 cloning	pRGK386
MSP366	gcgttttctaccggagcccagaaccaccagc	pMCS739 cloning	pRGK386
MSP367	gctggtggttctgggctccggtagaaaacgc	pMCS739 cloning	pRGK386
MSP368	gagttcgtaataggccgctgcggaaccgagc	pMCS736 cloning	pRGK386
MSP369	gctcggttccgcagcggcctattacgaactc	pMCS736 cloning	pRGK386
MSP370	cccagccgagttcggcataggcccatgcgg	pMCS740 cloning	pRGK386
MSP371	ccgcatgggcctatgccgaactcggctggg	pMCS740 cloning	pRGK386
MSP372	gccaccccagccgagtgcgtaataggcccatgcg	pMCS744 cloning	pRGK386
MSP373	cgcatgggcctattacgcactcggctggggtggc	pMCS744 cloning	pRGK386
MSP374	ccaccagccaccccaggcgagttcgtaataggc	pMCS741 cloning	pRGK386
MSP375	gcctattacgaactcgcctggggtggctggtgg	pMCS741 cloning	pRGK386
MSP376	ggcataaacgaggcgttttctgccggatcccagaaccaccagc	pMCS721 cloning	pRGK386
MSP377	gctggtggttctgggatccggcagaaaacgcctcgtttatgcc	pMCS721 cloning	pRGK386
MSP378	cccagaaccaccagccacccgcgccgagttcgtaataggccc	pMCS726 cloning	pRGK386
MSP379	gggcctattacgaactcggcgcgggtggctggtggttctggg	pMCS726 cloning	pRGK386
MSP380	cccagaaccaccagccagcccagccgagttcg	pMCS728 cloning	pRGK386
MSP381	cgaactcggctgggctggctggtggttctggg	pMCS728 cloning	pRGK386
MSP382	ccagaaccaccaggcaccccagccg	pMCS729 cloning	pRGK386
MSP383	cggctggggtgcctggtggttctgg	pMCS729 cloning	pRGK386
MSP384	cggatcccagaaccacgcgccaccccagccg	pMCS730 cloning	pRGK386
MSP385	cggctggggtggcgcgtggttctgggatccg	pMCS730 cloning	pRGK386
MSP386	ctaccggatcccaggcccaccagccacccc	pMCS731 cloning	pRGK386
MSP387	ggggtggctggtgggcctgggatccggtag	pMCS731 cloning	pRGK386
MSP388	cgaggcgttttctaccgcatcccagaaccaccagc	pMCS732 cloning	pRGK386
MSP389	gctggtggttctgggatgcggtagaaaacgcctcg	pMCS732 cloning	pRGK386
MSP390	cggcataaacgaggcggcttctaccggatcccag	pMCS742 cloning	pRGK386
MSP391	ctgggatccggtagaagccgcctcgtttatgccg	pMCS742 cloning	pRGK386
MSP392	gccacggcataaacgcggcgttttctaccgg	pMCS733 cloning	pRGK386
MSP393	ccggtagaaaacgccgcgtttatgccgtggc	pMCS733 cloning	pRGK386
MSP394	cggatcccagaacgcccagccaccccag	pMCS734 cloning	pRGK386
MSP395	ctggggtggctgggcgttctgggatccg	pMCS734 cloning	pRGK386
MSP396	cccagccgagttcgtaagcggcccatgcggaacc	pMCS722 cloning	pRGK386
MSP397	ggttccgcatgggccgcttacgaactcggctggg	pMCS722 cloning	pRGK386
MSP398	ccgagttcgtaataggcgcatgcggaaccgagc	pMSC767 cloning	pRGK386
MSP399	gctcggttccgcatgcgcctattacgaactcgg	pMCS767 cloning	pRGK386
MSP400	gccgagttcgtaatagcaccatgcggaaccgagc	pMCS769 cloning	pRGK386

MSP401	gctcggttccgcatggtgctattacgaactcggc	pMCS769 cloning	pRGK386
MSP402	cccagccgagttcgtaacaggcccatgcggaaccg	pMCS770 cloning	pRGK386
MSP403	cggttccgcatgggcctgttacgaactcggctggg	pMCS770 cloning	pRGK386
MSP404	cccagccgagttcgcaataggcccatgcgg	pMCS772 cloning	pRGK386
MSP405	ccgcatgggcctattgcgaactcggctggg	pMCS772 cloning	pRGK386
MSP406	ccaccccagccgaggcagtaataggcccatgcggaacc	pMCS773 cloning	pRGK386
MSP407	ggttccgcatgggcctattactgcctcggctggggtgg	pMCS773 cloning	pRGK386
MSP408	gccaccccagccgcattcgtaataggccc	pMCS774 cloning	pRGK386
MSP409	gggcctattacgaatgcggctggggtggc	pMCS774 cloning	pRGK386
MSP410	ccagccaccccagcagagttcgtaatagg	pMCS775 cloning	pRGK386
MSP411	cctattacgaactctgctggggtggctgg	pMCS775 cloning	pRGK386
MSP412	ccaccagccaccgcagccgagttcg	pMCS777 cloning	pRGK386
MSP413	cgaactcggctgcggtggctggtgg	pMCS777 cloning	pRGK386
MSP414	cccagaaccaccagccacaccagccgagttcg	pMCS925 cloning	pRGK386
MSP415	cgaactcggctggtgtggctggtggttctggg	pMCS925 cloning	pRGK386
MSP416	cccagaaccaccagcaaccccagccgagttcg	pMCS778 cloning	pRGK386
MSP417	cgaactcggctggggttgctggtggttctggg	pMCS778 cloning	pRGK386
MSP418	cggatcccagaaccagcagccaccccagc	pMCS916 cloning	pRGK386
MSP419	gctggggtggctgctggttctgggatccg	pMCS916 cloning	pRGK386
MSP420	cggatcccagaagcaccagccaccc	pMCS924 cloning	pRGK386
MSP421	gggtggctggtgcttctgggatccg	pMCS924 cloning	pRGK386
MSP422	ccggatcccagcaccaccagccacc	pMCS917 cloning	pRGK386
MSP423	ggtggctggtggtgctgggatccgg	pMCS917 cloning	pRGK386
MSP424	gcgttttctaccggatcgcagaaccaccagcc	pMCS926 cloning	pRGK386
MSP425	ggctggtggttctgcgatccggtagaaaacgc	pMCS926 cloning	pRGK386
MSP426	ggcgttttctaccggacaccagaaccaccagcc	pMCS918 cloning	pRGK386
MSP427	ggctggtggttctggtgtccggtagaaaacgcc	pMCS918 cloning	pRGK386
MSP428	cgaggcgttttctacgcaatcccagaaccaccagcc	pMCS919 cloning	pRGK386
MSP429	gctggtggttctgggattgcgtagaaaacgcctcg	pMCS919 cloning	pRGK386
MSP430	gcataaacgaggcgttttcgcacggatcccagaaccaccagcc	pMCS920 cloning	pRGK386
MSP431	ggctggtggttctgggatccgtgcgaaaacgcctcgtttatgc	pMCS920 cloning	pRGK386
MSP432	gcataaacgaggcgttgcataccggatcccagaaccaccagcc	pMCS921 cloning	pRGK386
MSP433	ggctggtggttctgggatccggtatgcaacgcctcgtttatgc	pMCS921 cloning	pRGK386
MSP434	ggcataaacgaggcgcattctaccggatcccagaaccacc	pMCS922 cloning	pRGK386
MSP435	ggtggttctgggatccggtagaatgcgcctcgtttatgcc	pMCS922 cloning	pRGK386
MSP539	ccccaccagccacggcataaacgagcagttttctaccggatccc	pMCS927 cloning	pRGK386
MSP540	gggatccggtagaaaactgctcgtttatgccgtggctggtgggg	pMCS927 cloning	pRGK386
MSP541	gcagtccccaccagccacggcataaagcaggcgttttctaccggatccc	pMCS929 cloning	pRGK386
MSP542	gggatccggtagaaaacgcctgctttatgccgtggctggtggggactgc	pMCS929 cloning	pRGK386
MSP552	ggcctattacgaagccggctggggtgg	pMCS738 cloning	pRGK386
MSP553	ccaccccagccggcttcgtaataggcc	pMCS738 cloning	pRGK386
MSP642	ggcatcgtgctcggttcctgctgggcctattacgaactcggc	pMCS1070 cloning	pRGK386
MSP643	gccgagttcgtaataggcccagcaggaaccgagcacgatgcc	pMCS1070 cloning	pRGK386
